# Injury deaths in Australian sport and recreation: Identifying and assessing priorities for prevention

**DOI:** 10.1371/journal.pone.0250199

**Published:** 2021-04-22

**Authors:** Lauren V. Fortington, Andrew S. McIntosh, Caroline F. Finch

**Affiliations:** 1 School of Medical and Health Sciences, Edith Cowan University, Joondalup, Australia; 2 School of Engineering, Edith Cowan University, Joondalup, Australia; Paracelsus Medizinische Privatuniversitat - Nurnberg, GERMANY

## Abstract

**Introduction:**

Sport and recreation is beneficial for health and wellbeing but comes with a probability of loss, including occasional fatal injuries. Following high-profile injury deaths in Australia, concerns are raised regarding the safety of sport participation. To understand the scale and scope of injury deaths, and identify potential prevention opportunities, the aim of this investigation was to describe the number and nature of fatal injuries in Australian sport and recreation.

**Methods:**

This is a retrospective cohort study of injury deaths reported between 1 July 2000 to 31 December 2019 using data from the National Coronial Information System, Australia. Unintentional deaths with an external cause, where the activity was recorded as sport and exercise during leisure time were included. Drowning deaths were excluded. Presented are the number and % of cases by age, sex, sport, broad cause and annual crude death rate (population).

**Results:**

There were 1192 deaths, averaging 63 per year. Deaths were mostly in males (84.4%), with the largest proportion in people aged 15–24 years (23.1%). Wheeled motor (26.9%) and non-motor (16.2%) sports accounted for the highest proportion of cases. The primary mechanism of death was most commonly blunt force (85.4%), followed by piercing/penetrating force (5.0%). The years 2001 and 2005 recorded the highest crude injury death rate (2001, n = 92, 0.47 per 100,000 population; 2005, n = 95, 0.47 per 100,000 population).

**Conclusions:**

On average, there is more than one injury death per week in a sport or recreation setting in Australia. Cases occurred in many sports and recreation activities, including those generally considered to be safe (e.g. individual athletic activities, team ball sports.) Detailed investigation of the coronial recommendations that are present within each case is now needed to understand and identify potential prevention opportunities.

## Introduction

Participation in sport and active recreation is part of Australia’s national health policy, which aims to have “more Australians, more active, more often” to capitalise on well-known health and social benefits [1]. Unfortunately, alongside these benefits there is the potential for direct adverse outcomes, such as injury, and indirect adverse outcomes, such as time loss from school or work, and reduction in participation [2]. Part of the risk management process is to weigh up the “*effect of uncertainty on objectives*” [3]. For this, high quality data are required to identify what types of adverse outcomes occur in a given activity, how often, and what impact they have.

The risks and uncertainties of injury from participation in sport and recreation are largely ignored in physical activity promotion strategies [4]. Yet injuries do occur when people are physically active, and these can be serious or fatal. In Australia, injury deaths in sport tend to gain considerable media and public attention [5]. Such cases cause widespread alarm and concern among the public with spokespeople invariably referring to them as a “freak event” or “just an accident” [6]. Referring to injury deaths as “one off occurrences” counters the ability to prevent similar situations from occurring in the future. Instead of avoidance, surveillance and regular public reporting of the number of such deaths, their causes and characteristics will give a better understanding in support of opportunities for prevention.

Identifying the size and scope of the fatal injury problem in sport and recreation is important because it determines the response required from sports organisation. As an example, Cricket Australia demonstrated a clear risk management pathway in response to a high-profile death from vertebral artery dissection (VAD). Cricket Australia assessed the incidence and mechanisms of VAD and participated in the revision of the British Standard for cricket helmets to introduce a neck guard [7–9]. Thus, knowledge of fatal injuries and their causes is needed to provide the basis for the identification of injury risk controls.

Internationally, the National Centre for Catastrophic Sports Injury Reporting (NCCSIR) in the USA has used data since the mid 1900’s to inform preventative policy decisions that have saved lives and reduced serious injury in a range of sports including American football, basketball, cheerleading and pole vaulting [10]. In Australia, sports deaths have been reported using State trauma registries [11], hospital admissions [12], media files [13], and insurance claims [14]. Each of these studies provides evidence that fatalities in community Australian sport and recreation occur. However, the specific rates and characteristics of these fatalities are largely unknown because they are not routinely reported at a national level or in a way that can be compared across regions or sports. This gap in reporting limits our potential to prevent these deaths from occurring. Therefore, to understand the scale and scope of injury deaths in Australian sport and recreation, this study aimed to establish the number, nature and activity for fatal injuries using data from the National Coronial Information System.

## Methods

Ethical approval was provided from Edith Cowan University (2019–00085), the Department of Justice and Regulation Human Research Ethics Committee (CF/16/8934) and the Western Australia Coronial Ethics Committee.

This retrospective cohort study used data from the Australian National Coronial Information System (NCIS), a repository for coroner-notified deaths, with an online retrieval system [15]. In Australia, deaths that are unexpected or of an unnatural cause are notified to a coroner for review and possible investigation. Available information in the NCIS includes demographic details of the deceased and supplementary reports (consisting of autopsy, toxicology, police and coronial investigation findings). The NCIS codes its data according to the Australian National Health Data Dictionary and the International Classification of External Causes of Injuries (ICECI). The information available about the activity at the time of death, specific cause and mechanism of the death mean that some insight to what occurred can be ascertained.

A search strategy was developed to identify all unintentional deaths with an external cause, where the activity was recorded as “sport and exercise during leisure time.” Cases that occurred during other activities were excluded (e.g. during employment a professional sportsperson). Cases were excluded if the intent was not unintentional (i.e. intentional self-harm or assault). Further, because drowning is extensively covered in the annual Royal Life Saving Australia National Drowning Reports [16], these cases were excluded using the mechanism of injury field (i.e. any of *“Drowning/ near drowning after intentionally entering water”; “Drowning/near drowning while in a body of water”;“Drowning/near drowning following a vehicle accident”;“Drowning/near drowning after being swept off rocks”;“Drowning/near drowning following fall into water”)* Using this approach, there is the possibility that some cases were retained where drowning was one reason listed in combination with another injury as the main (primary) mechanism.

Some cases take longer than others for investigative and administration processes to take place. Once complete, these are considered ‘closed’ and were available for analysis. Across the whole system (any year), the percentage of closed cases averages 90.9%. As at April 2020, a total of 72.0% of cases for 2018 were closed, dropping to 35.4% for 2019. Because of these time delays in case closure, the inclusion dates chosen for descriptive data and time trends differed.

For descriptive data, we were interested in overall patterns of injury, so all available cases in the NCIS were included to 31 December 2019. Descriptive analyses are presented for death counts by sex, age, activity category, sport and year.

For time trends, exposure time needed to be comparable so only deaths from 1 January 2001 to 31 December 2018 were included. The start date was chosen because the first year of collection did not begin until July 2000 (part year only). The end date was chosen because data from 2019 were incomplete at only 35% closure of cases.

The NCIS includes both registered and non-registered sport participants and participation data are not consistently available for sports and recreational activities across Australia. Thus, to consider changes over time, the national population in December of each year was extracted from the Australian Bureau of Statistics website [17].

A negative binomial with log link generalized linear model (NB-GLM) was used to compare annual population adjusted death rates. The number of deaths was the outcome with natural logarithm of the estimated resident population in Australia per year included as the offset variable. Statistical analyses were conducted with SPSS Version 25.0 with significance set at p < 0.05.

## Results

Between 2000 and 2019 inclusive, 1192 unintentional deaths from external causes associated with an activity code of sport and exercise during leisure were recorded in the NCIS. Demographic data of the deceased and the incident is presented in Table 1. Males accounted for most deaths (84.4%). The largest proportion of deaths occurred in people aged 15 to 24 years (23.1%). The median age of deceased was 36 years (range 3 to 97).

**Table 1:** Socio-demographic characteristics of sport and active recreation injury deaths in Australia, 2000–2019.

	n	%
**Sex**		
Male	1006	84.4
Female	186	15.6
**Age** (median, range)		
≤ 14	99	8.3
15–24	275	23.1
25–34	195	16.4
35–44	190	15.9
45–54	190	15.9
55–64	124	10.4
>65	119	10.0
**Country of residence of deceased**		
Australia	**1142**	**95.8**
International	**48**	**4.0**
*United Kingdom*	*11*	*0*.*9*
*United States of America*	*8*	*0*.*7*
*Germany*	*7*	*0*.*6*
*Japan*	*6*	*0*.*5*
*Other and unknown*^*#*^	*18*	*1*.*5*
**State of usual residence of deceased^**		
ACT	16	1.3
NSW	408	34.2
NT	46	3.9
QLD	234	19.6
SA	85	7.1
TAS	51	4.3
VIC	155	13.0
WA	147	12.3
Unknown* (includes international)	50	4.2
**State of incident in Australia^**		
ACT	7	0.6
NSW	438	36.7
NT	59	5.0
QLD	239	20.1
SA	82	6.9
TAS	60	5.0
VIC	135	11.3
WA	158	13.3
Unknown	14	1.2
**Year of Incident**		
2000–2004	335	28.1
2005–2009	378	31.7
2010–2014	287	24.1
2015–2019	192	16.1
**Season of Incident^**		
Summer	320	26.8
Autumn	324	27.2
Winter	253	21.2
Spring	295	24.7
**Activity type**		
Informal	865	72.6
Organised	308	25.8
Unspecified	19	1.6
Investigation*		
Inquest held	98	8.2
No inquest held	1093	91.7

*for protection of individual privacy, data are supressed where fewer than five cases (i.e. 1–4) are reported;

^percentages do not sum to 100 due to rounding;

#countries include Austria, Canada, Denmark, France, Netherlands, New Zealand, Norway, Saudi Arabia, Singapore, South Africa, Switzerland, United Arab Emirates

The sports categories with the highest proportion of deaths were wheeled motor (26.9%) and wheeled non-motor (16.2%) sports, followed by individual water sports (13.2%) and aero sports (9.7%) (Table 2). Males accounted for a larger proportion of cases across all but two sports: in equestrian, females comprised 64.9% of cases and in one sport (sport not specified as fewer than 5 cases) 50.0% of cases involved females. The primary mechanism of death was most commonly blunt force trauma (n = 1018, 85.4%), followed by piercing/penetrating force (n = 60, 5.0%). Other mechanisms (including thermal, threat to breathing and exposure, among others) accounted for fewer than 3% of all deaths each.

**Table 2:** Number of injury deaths by different types of sport and exercise during leisure time (n = 1192).

Activity category	n	%	% within activity category
Specific sport			
**Wheeled Motor Sports**	321	26.9	
Motorcycling	230	-	71.7
Motor car racing, drag racing	34	-	10.6
Riding an all-terrain vehicle (ATV)	28	-	8.7
Go-Carting	12	-	3.7
Unspecified or other	17	-	5.3
**Wheeled Non-Motored Sports**	193	16.2	
Cycling–road	118	-	61.1
Skateboarding	30	-	15.5
Cycling–mountain	21	-	10.9
Cycling—BMX	8	-	4.1
Unspecified or other	16	-	8.3
**Individual Water Sports**	157	13.2	
Swimming–recreational	44	-	28.0
Scuba diving	28	-	17.8
Surfing, boogie boarding	24	-	15.3
Fishing	23	-	14.6
Diving—cliff	5	-	3.2
Diving—unspecified	5	-	3.2
Snorkelling	5	-	3.2
Unspecified or other	23	-	14.6
**Aero Sports**	116	9.7	
Parachuting/sky diving	36	-	31.0
Hang gliding (unpowered)	20	-	17.2
Paragliding/parasailing (unpowered)	19	-	16.4
Gliding	18	-	15.5
Aerobatics	23	-	19.8
**Boating Sports**	82	6.9	
Unspecified boating sports	22	-	26.8
Water skiing	20	-	24.4
Jet skiing	18	-	22.0
Boating, sailing, yachting	10	-	12.2
Power boat racing	5	-	6.1
Unspecified or other	7	-	8.5
**Equestrian Activities**	77	6.5	100.0
Trail or general horseback riding	54	-	70.1
Rodeo	5	-	6.5
Unspecified or other	18	-	23.4
**Adventure Sports**	73	6.1	100.0
Bushwalking, hiking, tramping	32	-	43.8
Rock climbing	19	-	26.0
Abseiling, rappelling	6	-	8.2
Mountaineering	6	-	8.2
Unspecified or other	10	-	13.7
**Individual Athletic Activities**	55	4.6	-
Walking	27	-	49.1
Jogging/running	16	-	29.1
Unspecified or other	12	-	21.8
Target/Precision Sports*	26	2.2	-
Firearm Shooting	14	-	53.8
Golf	8	-	30.8
**Team Ball Sports**	25	2.1	-
Rugby league	8	-	32.0
Australian football	7	-	28.0
Unspecified or other	10	-	40.0
Ice or Snow Sports*	25	2.1	-
Alpine/downhill skiing	13	-	52.0
Snowboarding	8	-	32.0
**Combative Sports**	6	0.5	-
**Mixed sports** (*n = 8 groups specified with <5 cases per group)*	23	1.9	-
Missing information	13	1.1	-

For deaths resulting from blunt force trauma, the largest proportion occurred during wheeled motor sports (30.7% of all blunt force injury cases) and wheeled non-motor sports (18.6%), aero sports (11.3%) and equestrian activities (7.3%). Piercing/penetrating forces were most commonly in individual water sports (n = 42, 70.0%), 36 (86%) of which involved an animal. Most of the remaining piercing/penetrating cases were associated with target/precision sports (18.3%).

The years 2001 and 2005 recorded the highest rate per population of injury deaths (2001, n = 92, 0.47 per 100,000 population: 2005, n = 95, 0.47 per 100,000 population) (Table 3). There was an estimated 5.7% decrease per year in deaths (95% CI: 3.5 to 14% decrease), which was not statistically significant (S1 Fig).

**Table 3:** Number of injury deaths in Australian sport and recreation from 2000 to 2019, incidence rate per 100 000 population with 95% confidence interval (n = 1192).

Year*	n injury deaths	population	Deaths per 100 000 population (95%CI)
**2000**	40	19141036	0.21 (0.27–0.14)
**2001**	92	19386461	0.47 (0.57–0.38)
**2002**	59	19605441	0.30 (0.38–0.22)
**2003**	69	19827155	0.35 (0.43–0.27)
**2004**	75	20046003	0.37 (0.46–0.29)
**2005**	95	20311543	0.47 (0.56–0.37)
**2006**	63	20627547	0.31 (0.38–0.23)
**2007**	93	21016121	0.44 (0.53–0.35)
**2008**	58	21475625	0.27 (0.34–0.20)
**2009**	69	21865623	0.32 (0.39–0.24)
**2010**	70	22172469	0.32 (0.39–0.24)
**2011**	61	22522197	0.27 (0.34–0.20)
**2012**	46	22928023	0.20 (0.26–0.14)
**2013**	66	23297777	0.28 (0.35–0.21)
**2014**	44	23640331	0.19 (0.24–0.13)
**2015**	50	23984581	0.21 (0.27–0.15)
**2016**	39	24389684	0.16 (0.21–0.11)
**2017**	49	24773350	0.20 (0.25–0.14)
**2018**	37	25171439	0.15 (0.19–0.10)
**2019**	17	25464116	0.07 (0.1–0.04)

* population data as at December of each year, except 2019 where September data was used because December was not yet available at time of analysis.

## Discussion

This study presents a national summary of the scale and scope of the fatal injury problem in sport and recreation. The study reflects a very broad and disparate group of sports and recreational activities, as well as causes of death; from motorsports to team ball sports and shark attacks to ball impacts. There was an average of 63 deaths per year, equating to more than one per week. Considered together as a setting, injury deaths are surprisingly frequent. As expected, activities involving high levels of kinetic energy and/or high participant numbers were associated with a higher proportion of fatal cases in Australia. Wheeled sports, both motorised and non-motorised, were the most common activity category resulting in injury death. Wheeled sports include a broad range of specific sports/activities and most have potential for high-energy impacts and related injuries. A combination of human factors, vehicle factors, and environmental factors have been described previously for a small number of fatality cases in recreational wheeled sports (both motor and non-motor) [18]. Detailed analysis of the factors that contribute to wheeled sport deaths will be valuable step towards their future prevention.

Although we excluded cases of drowning, individual water sports still featured prominently in the cases, being the third most frequent activity category. Most of these deaths were attributable to piercing injury from animals, such as sharks and crocodiles. Such cases are known to occur with some regularity in Australian waters and they tend to be widely known through public media interest. Coroner recommendations for individual cases are noted in a report from 2011 [19]. As an example there are specific strategies included around use of repellents for sharks (e.g. devices that emit a deterrent electrical shield). The recommendations focus on making the physical environment safer and provide explicit guidance on what needs to happen and who needs to action it. Practical solutions such as the examples above will be valuable for other deaths that were identified in this study. Fewer than 8% of cases had a full inquest conducted. A review of the cause and mechanism of cases from within and across activity categories could help to identify opportunities to learn what occurred and what could be used to counter these cases.

There was a trend towards a reduction in the injury-death incidence rate over the 20-year period analysed. This finding is possibly confounded by the inclusion of only closed cases, with latter years noted to be lower in number which might suggest the Coroner investigation process is not yet completed. There have been several previous investigations into fatalities in sport however these have not been directly comparable at a national level. This is partly due to the use of different data sources. As examples, in Victoria, Australia, there were 614 fatalities relating to sport and recreation identified from a trauma registry between 2005–2015 [11]. An earlier study of the same region reported 48 deaths between 2001–2003 [20]. From national insurance claims data relating to Australian Football, an average of 3 deaths per year were reported [14]. Across all football codes, 34 deaths were identified through online media in a 6 year period across Australia [13]. Finally, in cricket, 274 fatalities relating to direct trauma were identified primarily using historical print media records [5]. These varied results make it difficult to draw meaningful conclusions on which sports are associated with the most fatalities. Complicating this further, different data sets also include different categorisations for ‘sport and recreation’. For example, the insurance claims data used a broad, settings-based inclusion of all club members and volunteers, as well as travel to/from club events, not just those who are actively playing the sport [14]. Some sports have established standards for collection of data relating to the more common, moderate severity injuries, promoted through consensus-based guidelines for recording and reporting injury, though fatalities are not explicitly noted in these [21]. Injuries, particularly in Australia, are often reported according to their external cause (e.g. falls), rather than the setting in which they occur. Agreement on the best way to record and report fatalities in sport and recreation would be valuable to support future investigations [22].

Media reports have been used for injury surveillance previously in Australia and continue to be part of catastrophic sports injury surveillance in the USA. Media reports can be useful to identify cases but are incomplete with respect to detail, particularly for the cause of death. Frequently, terms such as ‘collapsed’ are used to describe an event without confirmation of whether this was linked to the heart, head, internal organs or otherwise [13, 22]. The use of data from the NCIS in this study provided important detail and clarity on the cause of death which allowed differentiation of the injury deaths that were of interest.

Despite the advantages of a data system such as the NCIS, there are also limitations with the data sourced from it. The NCIS data are collected for coronial investigative purposes not specifically for surveillance or prevention, and limitations exist in the dataset. We chose to rely on the NCIS coded activity of “Sport and Recreation in Leisure time” to identify cases to enable a consistent approach to case ascertainment over time. However, sports participation could potentially also fall into other NCIS Activity categories and identifying these would require additional key word searches and manual case review. This would mean that searches based on this approach would be less reliable for future follow up of cases. The use of ICD Activity codes in other administrative data (e.g. Australian hospital records) has been shown to be unreliable because fields are missing or unspecified [23, 24]. It is not known whether this has a similar impact for the NCIS data. Another limitation is the potential for overlap in the different types of Activity that sports can contribute to. For example, noted earlier, cycling can be both an activity of transport and sport/recreation, therein posing difficulties with choices for activity classification which impact design of search strategy and case ascertainment. The influence of unclosed cases on the results is also a potential limitation as it relates to this study. However, with regular reporting, the opportunity exists to update the dataset as cases are closed. Delays in case closure can be attributed to various reasons (e.g. need for an inquest or investigations from additional organisations, such as workplace, police). The influence of this timing on this study potentially results in a small change to case numbers that impact trends.

## Conclusion

Serious and fatal injuries can, and do, occur when people are physically active. With strong promotion of the importance and value of sport and recreation in national health policy, awareness for the possibility of adverse outcomes, such as death, is a necessary component of risk management and safety planning. Due to the relative infrequency of fatal injuries and the limitations in how they are currently reported in research, it can be difficult to identify common patterns to inform injury prevention, safety practice and policy. This study establishes a baseline for the size and scope of fatal injuries that occur within Australian sport and recreation. There has been an overall decrease in fatal injury incidence rates over 2001–2018 but there is still a substantial number of fatalities. These injury deaths now need to undergo detailed investigation of the coronial recommendations that are present to understand and identify potential prevention opportunities.

## Supporting information

S1 Fig Injury death (by population rate) from sport and recreation from 2001 to 2018. (TIF)
