# Supplementary figures and images for: Injury deaths in Australian sport and recreation: Identifying and assessing priorities for prevention
Source: PLoS One. 2021 Apr 22;16(4):e0250199. doi: 10.1371/journal.pone.0250199 (PMC8062095; doi:10.1371/journal.pone.0250199)

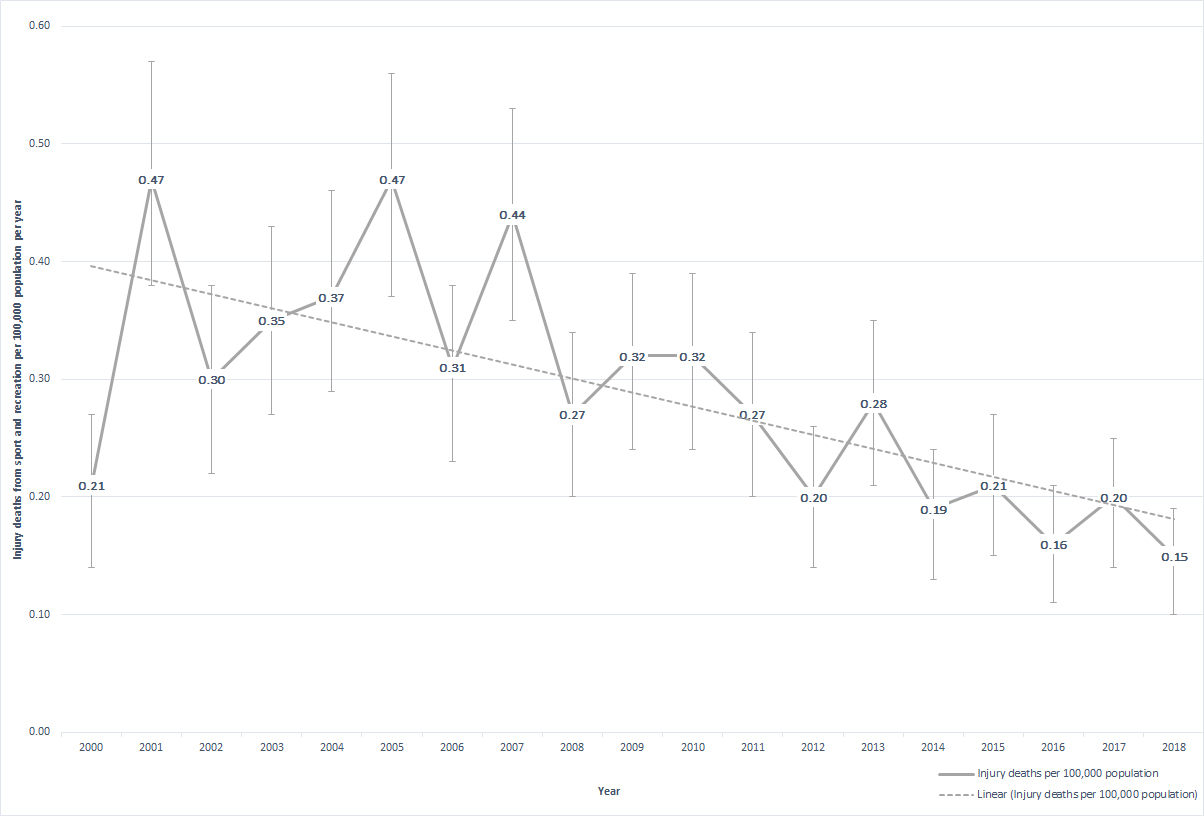

Supplement: S1 Fig — (TIF) [file pone.0250199.s001.tif]
